# Editorial: Responsible and robust evaluation for real-world recommendation and search systems

**DOI:** 10.3389/fdata.2026.1995917

**Published:** 2026-10-05

**Authors:** Alejandro Bellogín, Pablo Sánchez, Laura Sebastiá

**Affiliations:** 1 Escuela Politécnica Superior, Universidad Autónoma de Madrid, Madrid, Spain; 2 Instituto de Investigación Tecnológica (IIT), Universidad Pontificia Comillas, Madrid, Spain; 3 Escuela Técnica Superior de Ingeniería Geodésica, Cartográfica y Topográfica, Universitat Politècnica de València, Valencia, Spain

**Keywords:** large language models, multistakeholder fairness, real-world evaluation, recommender systems, search systems

## 1 Introduction

Both search and recommender systems are software tools that are used on a daily basis and influence a large number of decisions, affecting users, different stakeholders (companies, governments, organizations, etc.), and society as a whole (Ricci et al., 2022; Alonso and Baeza-Yates, 2024). Hence, as the evaluation of these systems is critical, it should go beyond performance prediction in traditional and/or artificial testing environments. Implemented real-world systems must deal with ambiguous data, temporal changes, conflicting interests among different stakeholders, and limits to generalization. All while they attempt to maintain stable performance despite changing circumstances.

Therefore, the evaluation must be robust, i.e., examining whether performance and behavior remain consistent under variable real-world environments, and responsible, i.e., ensuring that the objectives and indicators reflect the users and environments impacted by the system.

The four articles in this research topic explore some of these issues in four different domains: search query parsing, tourism recommender systems, career path prediction, and news moderation. Collectively, they show that the evaluation step cannot be viewed solely as the final stage of comparison between different proposals, but rather as a key component in the whole system design that reveals which system behaviors and trade-offs should be prioritized.

Baysan et al. propose an LLM-as-a-Judge framework using different versions of Gemini to evaluate structured query parsing outputs before live A/B-testing is available. Their framework supports three evaluation methodologies: Pointwise (which assigns a numerical score based on the evaluation criteria), Pairwise (used for comparing two parsed outputs), and Pass/Fail (binary classification). The reported results show high agreement with human judgments in both a small cleaned dataset and a large real-world dataset. However, the authors also report difficulties with hallucinated outputs related to categories in their error analysis. This work shows the scalability of the proposed LLM-based evaluation framework, while the authors also state that LLM evaluators should be validated using human evaluation to ensure reliability.

Müllner et al. analyze in their survey what algorithmic fairness can learn from tourism management. After their initial processing, they analyze 44 articles, 24 from tourism management, and 20 from computer science. Their work compares contextual, qualitative, and participatory approaches, which are common in tourism research, with the more restrictive formal criteria typically used in computer science. Although computer science and tourism management analyze the concept of fairness from different perspectives, they argue that closer interdisciplinary collaboration is critical to develop responsible recommender systems. The authors argue for the use of tourism research to expand the definitions of algorithmic fairness, identify different stakeholders, the trade-offs that occur in the real world, include participatory processes in system design, and transform qualitative objectives, such as reducing overtourism, into measurable indicators aligned with broader sustainability and equity goals.

Senger et al. compare linear transformations, multilayer perceptron (MLP), LSTM, and large language models (LLMs) approaches to career path prediction in two datasets (DECORTE and KARRIEREWEGE+), and with different input representations. Moreover, the authors also measure the effect of the incorporation of synthetic data in the performance of the models. In the reported results, when job titles are combined with descriptions, results are generally better than when using title-only inputs. In most cases, their MLP proposal achieves the best performance, setting new state-of-the-art scores, while LSTMs obtain good results when the experiment involves longer career histories. The performance obtained by LLMs is highly affected by fine-tuning, grounding, and alignment of the dataset. Finally, their analysis also shows that even if synthetic augmentation may increase diversity, it may also introduce semantic distortions.

Waterschoot and van den Bosch propose a group recommender system implemented by different classifiers that helps moderators select which comments to highlight on news platforms. Since selecting comments manually requires significant resources and attention from moderators, they propose a group recommender system that uses classifiers that integrate both contextual relevance and user history to rank the comments. They tested their approach in unobserved discussions from 2020 and 2023, showing robustness against a variety of topic factors. Although the results obtained by the best model have been validated by experts, the authors believe the system is designed to support moderators, not to replace them, so ultimately the moderators have the final say.

## 2 Discussion

After analyzing the works in the collection, three concepts emerge. First, the evaluation settings should closely resemble a real-world environment through temporal testing, different datasets and representations, query patterns reflecting real browsing behavior, and an explicit analysis of the different stakeholder needs. Second, human expertise in the domain remains critical: moderators' evaluations, professional taxonomies, experts' annotations, and stakeholder participation help in determining what is considered suitable, accurate, or fair by the system in each specific domain. Third, there is no single aggregate metric that can capture all the relevant behavior that the system should analyze. Classification and ranking metrics should be complemented with experiments performed using different groups of users, by computing reliable statistics, providing qualitative error analysis, and making stakeholder-oriented testing to show complementary perspectives in real-world applications.

The evaluation should then be considered as a continuous social and technical process based on the stakeholders involved in the system life-cycle. It is critical to define which results and interests are important according to the different affected stakeholders. Evaluation protocols should include realistic scenarios that examine potential failures that may occur under human supervision. In addition, assumptions and limitations of the studies must be clearly stated so that they can serve as the basis for responsible decision-making processes, showing the ambiguity and disagreements behind the overall scores.
